# Supplementary figures and images for: Analytic approaches to clinical validation of results from preclinical models of glioblastoma: A systematic review
Source: PLoS One. 2022 Mar 1;17(3):e0264740. doi: 10.1371/journal.pone.0264740 (PMC8887747; doi:10.1371/journal.pone.0264740)

**S1 Figure. Common analytic strategy used by included studies.**

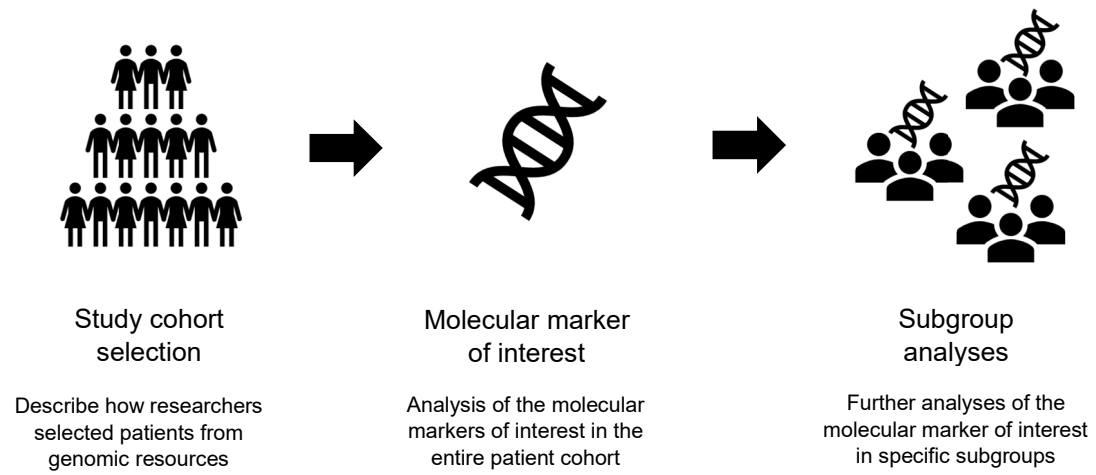

Supplement: S1 Fig — (PDF) [file pone.0264740.s002.pdf]
