# Supplementary material for: Analytic approaches to clinical validation of results from preclinical models of glioblastoma: A systematic review
Source: PLoS One. 2022 Mar 1;17(3):e0264740. doi: 10.1371/journal.pone.0264740 (PMC8887747; doi:10.1371/journal.pone.0264740)
Supplement: S1 Table — Data type: A = RNA microarray only, B = RNA microarray and miRNA microarray, C = RNA sequencing only, D = RNA microarray and RNA sequencing, E = miRNA microarray only, F = RNA sequencing, RNA microarray and miRNA microarray, G = RNA sequencing and miRNA microarray, H = RNA microarray and DNA methylation, I = RNA sequencing, RNA microarray and DNA methylation, J = Unspecified. If a study used a data source but not specified the number of patients, the column for data source would be “Yes [NS]” indicating number of patients not specified. (PDF) [file pone.0264740.s003.pdf]

S1 Table. Characteristics of 58 included studies

Author, year of publication	Cohort selection described	Data source [number of patients]		Data type	Molecular marker(s) or set of variables of interest				Survival analysis	
		TCGA [number]	CGGA [number]		One	More than one	Set of markers	Set of markers and clinical variables	Univariable	Multivariable
Alvarado, 2016	No	Yes [NS]	No [--]	B	No	Yes	Yes	No	Yes	No
Bayin, 2016	No	Yes [160]	No [--]	C	Yes	No	No	No	Yes	No
Cai, 2020	Yes	Yes [103]	Yes [83]	C	No	No	Yes	Yes	Yes	Yes
Delic, 2014	No	Yes [519]	No [--]	B	No	Yes	No	No	Yes	Yes
Deng, 2017	No	Yes [540]	No [--]	A	Yes	No	No	No	Yes	Yes
Erhart, 2018	No	Yes [1022]	No [--]	B	No	No	Yes	No	Yes	No
Erhart, 2019	No	Yes [660]	No [--]	A	No	No	Yes	No	Yes	No
Genovese, 2012	No	Yes [290]	No [--]	B	No	Yes	No	No	Yes	No
Guo, 2018	No	Yes [169]	No [--]	A	Yes	No	No	No	Yes	Yes
Haapa-Paananen, 2013	No	Yes [308]	No [--]	B	No	Yes	No	No	Yes	No
Hasan, 2019	No	Yes [265]	No [--]	A	Yes	No	No	Yes	Yes	Yes
Ho, 2017	No	Yes [694]	No [--]	F	No	Yes	Yes	No	Yes	Yes
Holmberg Olausson, 2014	No	Yes [NS]	No [--]	A	Yes	No	No	No	Yes	No
Hong, 2016	Yes	Yes [170]	No [--]	E	Yes	No	No	Yes	Yes	No
Hu, 2012	No	Yes [425]	No [--]	A	No	Yes	No	No	Yes	No
Hua, 2020	No	Yes [206]	No [--]	A	Yes	No	No	No	Yes	No
Jarboe, 2012	Yes	Yes [192]	No [--]	H	Yes	No	No	Yes	Yes	Yes
Kinker, 2016	No	Yes [152]	No [--]	C	Yes	No	Yes	No	Yes	Yes
Klopfenstein, 2019	Yes	Yes [481]	No [--]	A	No	Yes	Yes	No	Yes	Yes
Kuang, 2018	No	Yes [540]	No [--]	A	Yes	No	Yes	No	Yes	No
Kudo, 2020	No	Yes [525]	No [--]	A	No	Yes	No	No	Yes	Yes
Li, 2018	Yes	Yes [92]	No [--]	C	No	Yes	Yes	No	Yes	No
Li, 2019	No	Yes [NS]	No [--]	D	Yes	No	No	No	Yes	No
Liu, 2019	No	Yes [565]	No [--]	B	No	Yes	No	No	Yes	Yes
Luedi, 2017	No	Yes [515]	No [--]	A	No	No	Yes	No	Yes	Yes

Luedi, 2018	No	Yes [520]	No [--]	A	No	No	Yes	No	Yes	No
Mao, 2015	No	Yes [473]	No [--]	A	Yes	No	No	No	Yes	Yes
Marziali, 2016	No	Yes [251]	No [--]	C	No	No	Yes	No	Yes	Yes
Mega, 2020	Yes	Yes [569]	No [--]	A	No	Yes	Yes	No	Yes	Yes
Mehrian-Shai, 2015	Yes	Yes [210]	No [--]	A	No	Yes	No	No	Yes	No
Mikheev, 2018	No	Yes [525]	No [--]	A	Yes	No	No	No	Yes	No
Nduom, 2016	No	Yes [149]	No [--]	C	No	Yes	Yes	No	Yes	Yes
Okura, 2016	Yes	Yes [528]	No [--]	A	Yes	No	No	No	Yes	No
Polonen, 2019	No	Yes [520]	No [--]	D	Yes	No	No	No	Yes	Yes
Pandya, 2019	No	Yes [142]	No [--]	A	No	Yes	No	No	Yes	No
Pangeni, 2018	Yes	Yes [348]	No [--]	A	Yes	No	No	No	Yes	No
Paul, 2018	Yes	Yes [172]	No [--]	G	No	Yes	Yes	No	Yes	Yes
Pollak, 2017	Yes	Yes [525]	No [--]	A	No	Yes	No	No	Yes	No
Qiu, 2013	Yes	Yes [480]	No [--]	B	Yes	No	No	No	Yes	No
Rowther, 2016	No	Yes [596]	No [--]	A	No	No	Yes	No	Yes	No
Safaee, 2013	No	Yes [212]	No [--]	A	Yes	No	No	No	Yes	No
Sana, 2018	Yes	Yes [485]	No [--]	E	No	No	Yes	No	Yes	Yes
Sathyan, 2015	Yes	Yes [353]	No [--]	B	No	Yes	Yes	No	Yes	Yes
Shahar, 2017	No	Yes [414]	No [--]	A	No	Yes	Yes	No	Yes	Yes
ShaRNA, 2017	No	Yes [39]	No [--]	D	No	Yes	No	No	Yes	No
Shi, 2015	No	Yes [476]	No [--]	B	No	Yes	Yes	No	Yes	Yes
Shugg, 2020	Yes	Yes [152]	No [--]	A	No	Yes	No	No	Yes	No
Stegen, 2015	No	Yes [NS]	No [--]	C	Yes	No	No	No	Yes	No
Wang, 2019	No	Yes [153]	No [--]	A	Yes	No	No	No	Yes	No
Wen, 2020	No	Yes [264]	No [--]	A	Yes	No	No	No	Yes	No
Xavier-Magalhaes, 2018	Yes	Yes [554]	No [--]	I	Yes	No	No	Yes	Yes	Yes
Xu, 2017	No	Yes [NS]	Yes [NS]	A	Yes	No	No	No	Yes	Yes
Yadav, 2009	No	Yes [407]	No [--]	B	Yes	No	No	No	Yes	Yes
Yeung, 2020	No	Yes [573]	No [--]	J	Yes	No	No	No	Yes	No

Yi, 2018	No	Yes [540]	No [--]	J	No	Yes	No	No	Yes	No
Zeng, 2018	No	Yes [NS]	No [--]	F	No	Yes	No	No	Yes	Yes
Zhai, 2017	No	Yes [172]	No [--]	D	Yes	No	No	No	Yes	Yes
Zhang, 2017	No	Yes [607]	No [--]	J	Yes	No	No	No	Yes	No

Data type:

A = RNA microarray only

B = RNA microarray and miRNA microarray

C = RNA sequencing only

D = RNA microarray and RNA sequencing

E = miRNA microarray only

F = RNA sequencing, RNA microarray and miRNA microarray

G = RNA sequencing and miRNA microarray

H = RNA microarray and DNA methylation

I = RNA sequencing, RNA microarray and DNA methylation

J = Unspecified

If a study used a data source but not specified the number of patients, the column for data source would be “Yes [NS]” indicating number of patients not specified.
