# Supplementary material for: Analytic approaches to clinical validation of results from preclinical models of glioblastoma: A systematic review
Source: PLoS One. 2022 Mar 1;17(3):e0264740. doi: 10.1371/journal.pone.0264740 (PMC8887747; doi:10.1371/journal.pone.0264740)
Supplement: S2 Table — Molecular markers ordered alphabetically accompanied with the location of analysis in the original manuscript. U = univariable survival analysis; M = multivariable survival analysis; ▲ = positive association i.e. higher levels of the molecular marker associated with better survival and p<0.05; ▼ = negative association i.e. lower levels of molecular marker associated with worse survival and p<0.05; □ = statistical significance not demonstrated (p≥0.05). (PDF) [file pone.0264740.s004.pdf]

S2 Table. Refences to specific analyses extracted for comparison of results on molecular markers

Molecular marker	Author	Analysis type	Association	Location of analysis in manuscript
CXCL14	Zeng 2018	U	▼	Figure 3K reporting p=0.02809
		M	□	Supplementary Table 2 reporting HR 1.04 p=0.352
EGFR	Kuang 2018	U	▼	Figure 7, top survival curve in left panel reporting p=0.0009
	Li 2018	U	□	Figure 4A lower panel reporting p=0.216
HOTAIR	Xavier-Magalhaes 2018	U	▼	Figure 4B p=0.032
		M	▼	Supplementary Table 2 p=0.036
IDO1	Zhai 2017	U	▼	Figure 2C right panel, p<0.05
		M	▼	Table 1, HR 1.82 (95% CI 1.17-2.81) p=0.0076
IL-8	Hasan 2019	U	▼	Figure 2B top left panel p=0.0112
		M	▼	Legend of Figure 2B, HR 1.07, 95% CI 1.01-1.14), p=0.0467
MARCKS	Jarboe 2012	U	▲	Figure 6B, p=0.0002
		M	▲	Supplementary Table 2, HR 0.21 (95% CI 0.09-0.48), p=0.0002
miR-17-5p	Zeng 2018	U	▲	Figure 6D reporting p=0.0006
		M	□	"After incorporating the age and molecular subtype factors, the analysis indicated that miR-17-5p value was not an independent prognostic marker for the overall survival of GBM patients"
miR-181d	Genovese 2012	U	□	Supplementary Table 6 on page 606 of supplementary PDF file, p=0.46
	Ho 2017	U	□	Figure 4D, p=0.067
miR-34a	Genovese 2012	U	▼	Figure 3D reporting p=0.0154
		M	▼	Supplementary Table 7 on page 607 of supplementary PDF file reporting p=0.0016
NTN4	Hu 2012	U	▲	Figure 3B, p<0.05
	Li 2018	U	□	Figure 4B, lower panel, p=0.753
PD-L1	Nduom 2016	U	▼	Figure 4A, p=0.231
		M	▼	Text: "HR 1.54, 95% CI 1.05-2.28, p=0.0231"
POSTN	Mega 2020	U	▼	Table 2, HR 1.35, 95% CI 1.11-1.64, p=0.002
	Liu 2019	U	▼	Figure 7h, p=0.0003
	Mega 2020	M	▼	Table 2, HR 1.37, 95% CI 1.06-1.77, p=0.017
SFRP1	Delic 2014	U	▲	Figure 7B, p<0.05
		M	▲	Table 1, HR 0.782 (95%CI 0.642-0.953), p=0.015
Sox2	Sathyan 2015	U	▲	Figure 6C, 0=0.0307
		M	□	Text "…after correcting for age and KPS, Sox2 lost significance (p=0.0964)"
SRGN	Mega 2020	U	▼	Table 2, HR 1.63, 95% CI 1.19-2.23, p=0.002
		M	□	Table 2, HR 1.29, 95% CI 0.83-2.00, p=0.263

Molecular markers ordered alphabetically accompanied with the location of analysis in the original manuscript. U = univariable survival analysis; M = multivariable survival analysis; ▲ = positive association i.e. higher levels of the molecular marker associated with better survival and p<0.05; ▼= negative association i.e. lower levels of molecular marker associated with worse survival and p<0.05; □ = statistical significance not demonstrated (p≥0.05)
