# Supplementary material for: Analytic approaches to clinical validation of results from preclinical models of glioblastoma: A systematic review
Source: PLoS One. 2022 Mar 1;17(3):e0264740. doi: 10.1371/journal.pone.0264740 (PMC8887747; doi:10.1371/journal.pone.0264740)
Supplement: S1 File — (PDF) [file pone.0264740.s006.pdf]

S1 Supporting Information

Eligibility criteria

Inclusion:

- Patients (≥ 18 years old) diagnosed with non-recurrent histopathologically confirmed glioblastoma according to WHO classification
- Studies which utilised TCGA/CGGA resources or included TCGA/CGGA patients with other patients
- Studies reporting overall survival
- Studies reporting on association between genetic or molecular markers with survival
    - Gene expression, variants, methylation

Exclusion:

- Case reports, reviews, editorials
- Studies which only report their own patient cohort and does not include TCGA/CGGA resources
- Studies which report results only for patients (< 18 years old)
- Studies which include patients < 18 years old and have not separated their data from those >18
- Studies that reported progression free survival only
- Studies including patients with recurrent glioblastoma only
- Patients without a histopathological confirmed glioblastoma diagnosis according to WHO classification
