# Supplementary material for: Analytic approaches to clinical validation of results from preclinical models of glioblastoma: A systematic review
Source: PLoS One. 2022 Mar 1;17(3):e0264740. doi: 10.1371/journal.pone.0264740 (PMC8887747; doi:10.1371/journal.pone.0264740)
Supplement: Attachment — Submitted filename: Response to reviewers_PLOS One_v2.docx [file pone.0264740.s008.docx]

**Response to reviewers’ comments [PONE-D-21-32960]**

**Editor**

The authors claim that preclinical models of glioblastoma do not include sufficient details to compare different studies, particularly when the results are inconsistent. Clearly different results are to be expected when different studies look at different cohorts, e.g. different molecular subtypes. The question is, are these differences clear enough in the manuscript. I am concerned that these details are indeed clearly described in their manuscripts but the authors have not read these studies in sufficient detail. See Reviewer 1 comments.

**Response**: Thank you for highlighting this concern about our manuscript. It is correct that some of the included studies contain descriptions about their cohort selection. We have now included a table of study characteristics in Supplementary Table 1 and have provided a summary of the proportion of studies reporting their cohort selection in the results section. Frequently, researchers are interested in the association between a molecular marker and survival. Although studies may set out to investigate this association in a patient subgroup with a specific molecular subtype, it is common that studies also report the association in the entire cohort *before* conducting subgroup analyses. The figure below represents this schema:

Study cohort selection

Molecular marker of interest

Subgroup analyses

Describe how researchers selected patients from genomic resources

Analysis of the molecular markers of interest in the entire patient cohort

Further analyses of the molecular marker of interest in specific subgroups

As you and the other reviewers suggested, we cannot compare results from different subgroup analyses. Our data collection procedure therefore retrieved not only the final results presented in the studies, but also results from any interim analyses performed. For examples, two studies may describe the same cohort selection from TCGA and both investigating the same molecular marker. But they may differ in subgroup analyses. Results reported on the association between the molecular marker and survival in the overall cohort (and not the subgroup analyses) should be comparable. Our results about inconsistencies relate to comparable cohorts not specific to molecular subtypes of tumours. In the example Reviewer 1 gave, both Kuang *et al*. (<https://doi.org/10.1111/cas.13707>) (Figure 7, top survival curve in left panel reporting p=0.0009) and Li *et al*. (<https://doi.org/10.1186/s12885-018-5056-4>) (Figure 4A lower panel reporting p=0.216) presented survival curves of patients with high or low EGFR using the Kaplan-Meier method in their interim analyses. There were no specific cohort selection criteria that preclude comparison since they included all available patients and used log-rank test to compare survival functions. The difference between these studies was that Kuang *et* al. used RNA microarray data and Li *et* al. used RNA-Seq data. The association should be consistent across different data type, but this was not the case.

We have clarified this in our methods section (second paragraph under “Data extraction and data items” and penultimate sentence under “Summary statistics”) and provided further information in the supplementary materials (Supplementary Table 2 specifies the analysis used for comparison).

The “Study characteristics” section should be expanded to describe in detail differences in cohort selection, analysis method, etc. This section should be structured help answer the above question.

**Response**: Thank you for this suggestion. We have expanded on our description of these aspects. There is also an additional table in the Supplement with further study characteristics. We believe these changes have improved the clarity of our review in answering the question above.

The authors should also suggest a set of characteristics that should be listed in the Methods section so that different studies can be easily compared.

**Response**: We considered recommending a list of items to be reported in the Methods section of similar studies. In our discussion, we decided not to include this. These studies have different aims. Some may only be interested in determining the association between a molecular marker and survival, while others set out to assess how molecular markers may predict survival. These are related but different study designs require specific reporting standards. We have signposted the relevant guidelines in our discussion section i.e. STROBE, TRIPOD, REMARK guidelines. Developing a new set of items for reporting would lead to duplication and recommendations that cover all types of studies would be too broad.

In its current form this study does little more than state the obvious, albeit with specific examples. Additional rigor and detail are required for this paper to be acceptable for publication.

**Response**: We recognise that our results are not novel, however we disagree that the results are well-known to researchers in the field. There are three main points in our manuscript: (1) that there is need for clarity in cohort selection; (2) that there are inconsistencies in results from comparable studies; (3) that there are confounding effects from clinical variables, which are often not considered. If these observations are well-known then we would expect that researchers would better account for them in their research.

Our results demonstrate that preclinical glioblastoma studies using genomic repository data for clinical validation have not adequately addressed these three seemingly obvious points. This distracts research priorities and leads to suboptimal study design. The opportunity here is to enhance reproducibility and clinical relevance of preclinical studies by considering the three main points raised in our manuscript. We have edited our manuscript to emphasise this.

**Reviewer #1:**

The authors reasoned that preclinical model for association testing between molecular markers and overall patient survival should include more complete patient characteristics. This was studied in the TCGA glioblastoma cohorts in RNA microarray data. They concluded that preclinical GBM studies with incomplete patient characterization resulted in inconsistent results.

Comments:
1. CCGA should be mentioned in the abstract. Also, RNA sequencing and RNA microarrays were assessed in this analysis.

**Response**: We have now added CGGA in the abstract. We have not specified the data types in our abstract because our review did not restrict to studies using any genomic data type. Inclusion into this review was based on using TCGA or CGGA for clinical validation of results from preclinical studies. We have clarified this in the Methods section (Methods section under “Eligibility criteria”).

2. The results from Table 2 should provide context and be modified e.g. EGFR was reported to not have inconsistent results because Kuang et al. reported a Kaplan-Meier survival analysis using data from The Cancer Genome Atlas dataset which indicated that the expression of Cx43 significantly improved the prognosis of GBM patients who express EGFR while Li et al., showed that neither EGFR nor NTN4 expression significantly correlated with patient survival after TMZ treatment, while co-expression of EGFR/NTN4 predicts poor patient survival. These two studies can not be compared because they are looking at specific subgroups within their pool of patients which was defined by other molecular markers.

**Response**: Thank you for your helpful comment to clarify our table. We have addressed to this concern in our response to the comments from the editor. To avoid repetition in this document, please refer to our response above.

Also, Genovese et al. integrated data to produce a network model with molecular subtypes of GBM and functional genomic screen to determine associations with patient survival. Each pool of patients is sub categorized into specific glioblastoma molecular subtypes.

**Response**: This study by Genovese *et al*. is interesting as a piece of work with an analytic approach that considers molecular subtypes characterised in the seminal work by TCGA (<https://doi-org.ezproxy.is.ed.ac.uk/10.1016/j.cell.2013.09.034>). The study explored the role of miR-34a in different molecular subtypes of glioblastoma. However, they also presented results from the overall cohort of 271 patients. The univariable analysis is presented in Figure 3D showing a Kaplan-Meier curve of patients whose tumour had high or low miR-34a levels. The associated log-rank test had p=0.0154. The authors also examined the confounding effects of clinical variables, but these results are presented in Supplementary Table 7 (page 607 of supplementary PDF file) – there they reported multivariable Cox regression on survival with variables including miR-34a, primary vs secondary glioblastoma, received chemotherapy or not, and sex. In this multivariable analysis, the hazard ratio for miR-34a was 1.2 with p=0.0016. Data presented in Table 2 of our manuscript reflect these analyses instead of the subgroup analyses. Again, we hope our clarification in our methods section (second paragraph under “Data extraction and data items” and penultimate sentence under “Summary statistics”) and the supplementary materials (Supplementary Table 2) have resolved this concern.

The authors make a valid and important point that translational studies in GBM should increase their transparency and consider patient characteristics when publishing their findings. I suggest that the authors modify their results after reviewing the manuscripts in Table 2.

**Response**: Thank you for your kind comments about the importance of this work. We have carefully reviewed the included studies and our tables, and made changes to our manuscript to improve the clarity of our review process and results.

**Reviewer #2**:

In ' Analytic approaches to clinical validation of results from preclinical models of glioblastoma: a systematic review,' the authors searched on Embase and Medline for glioblastoma studies using preclinical models that also utilized data from TCGA and/or CGGA to validate the association between molecular markers and overall survival. Out of the 58 eligible studies from 1,751 non-duplicate records, they sorted out a total of 126 molecular markers that were reported. And of the 15 molecular markers reported and analyzed in more than one study, five showed discrepancies among studies. The authors argued that these inconsistent results are probably due to different analytic approaches of survival analyses used in different studies, thus concluded that increasing the transparency in reporting and the use of analytic methods that adjust for clinical variables could improve the reproducibility of these findings. This is a short but interesting study, even though the finding – discrepancies among studies – is not surprising. The authors discussed a few possibilities to improve the reproducibility of studies, such as using multivariable analysis considering multiple clinical variables, providing the query data and code-based commands used in the study. I am not sure why the authors picked glioblastoma. However, suppose they can expand the review on other cancer types with data available from TCGA. In that case, it might be as well useful to discuss about how to filter out studies with unreliable results.

**Response**: Thank you for the positive comments. We chose glioblastoma because since 2005 no novel therapies have been effectively translated into the clinic, despite promising data from a plethora of pre-clinical studies. There are multiple barriers to translational glioblastoma research, many of which relate to challenges in developing clinically relevant preclinical models and effective drug screening. In this review, we focused on methodological aspects of preclinical studies aiming specifically for translational value. We have edited our introduction to explain this more clearly to contextualise results of our review. Findings from our review should encourage researchers in this field to maximise reproducibility and improve analytic approaches. The findings will likely also be applicable to other cancers.

**Reviewer #3**:

The authors have done a commendable work of selecting relevant studies from a large corpus to assess consistency and proper utilization of patient characteristics in those studies.

**Response**: Thank you for your positive comment.

A brief review/overview of survival analysis with different variables can set the proper context for this review work. This review paper is missing the necessary background. The results are not explicitly tied to the studies under review. The authors should state the findings from the studies while presenting their conclusion.

**Response**: Thank you for this comment. Clinically, it is widely understood that adjusting for clinical variables when analysing patient data for survival is crucial when trying to find the true relationship between a marker with survival, as no two patients are the same. Therefore Kaplan-Meier analyses are inappropriate in this context. To more clearly set the context of this review, we have expanded on types of survival analyses and utilisation of clinical variables in the background.

Other comments:

Move the citation number before the full stop throughout the document. ...developing novel therapies.[5] >> ...developing novel therapies[5].

**Response**: We have reviewed all citations and edited accordingly.

The authors have chosen studies for their review that included data from TCGA and CGGA but haven't considered ICGA. Is there a reason for omitting the last repository?

**Response**: Thank you for raising this. The Indian Cancer Genomic Atlas (ICGA), to the best of our knowledge, has submitted their documentation for ethics committee approval in March 2021 (<https://www.icga.in/about-icga-project.html>). The first pilot study is based in breast cancer rather than glioblastoma. No glioblastoma genomic data is available from ICGA at present. So, we did not include ICGA. We do anticipate ICGA to be a valuable resource to validate findings from the predominantly white population in TCGA, as well as to drive discovery science in cancer research.

It was not clear what the authors meant by "stratified by the data sources" on page 5, the first paragraph. A structured demonstration of stratified results would be helpful.

**Response**: “Data resources” refer to either TCGA or CGGA. To anticipate studies using data from both TCGA and CGGA resources, we only included studies that separately reported results for each of these resources. This is to allow for comparison between studies. We have edited this section to clarify this (Methods section under “Eligibility criteria”). It now reads: “*Studies using both TCGA and CGGA were eligible if they had separately reported results for TCGA and CGGA*”.

The authors haven't listed the screening process in detail. I would love to know the different criteria the first two reviewers considered for accepting or rejecting a paper. This will also give more credibility to the selected studies.

**Response**: Both reviewers agreed on a list of inclusion and exclusion criteria prior to screening to minimise the risk of bias and inconsistency. Conflicting results were reviewed by an independent third reviewer. We have described this in the “eligibility criteria” subsection under the Methods section. For clarity, we now have added our point-by-point inclusion and exclusion criteria to the supplementary materials (Supplementary Materials – “Eligibility criteria”).

Please cite Covidence.

**Response**: We have referenced Covidence now.

"A set of molecular markers was defined by a grouping..." -- what is this grouping and what is the process and significance of this grouping?

**Response**: A set of molecular markers were defined by the studies themselves when they looked for associations between survival and a group of >1 molecular markers rather than analysing markers individually. For our review, it was important to distinguish analyses of individual vs groups of markers as no two groups are the same and results are therefore incomparable with other studies. We have amended our definition in the methods section (Methods section under Data extraction and data items). It now reads: “*A set of molecular markers was defined by the analysis of >1 molecular markers together.*”

Page 6, "Quality assessment", please write the first sentence to clarify. Please quantify/quantitatively define the measures of quality relating to the risk of bias.

**Response**: We have amended this section to clarify our quality assessment (Methods section under “Quality assessment”). Because the aim of this review is not to summarise or meta-analyse results from the included studies, we did not produce a quantitative risk of bias that would normally be available from a systematic review.

The authors claimed the multivariable analyses are better than univariable. However, there is no direct proof to support the claim in this context.

**Response**: We agree that multivariable analyses are not definitively better than univariable analyses. While multivariable analyses controlling for confounders in the setting of an observational study is appropriate, univariable analysis of the primary outcome in a randomised controlled trial of an intervention would be appropriate. Our message in this manuscript is about consideration of clinical variables in survival analyses. The importance of this stems from confounding. Age, functional status, treatment received, and extent of resection are known factors associated with survival in patients with glioblastoma. As such, these can have confounding effects on the association between a molecular marker and survival. Multivariable analyses can be used to account for these confounders. It is based on this that we recommend multivariable rather than univariable survival analyses. We have provided more information and references about these in our introduction (second paragraph in Introduction) and discussion section to clarify our narrative (Discussion section under “Confounding effects of clinical variables").

Page 7, Reproducibility section: It's unclear why the 12 studies that did not perform a multivariable survival analysis should have the same number of patients included.

**Response**: The number of patients from which data is available depends on several factors. First is the genomic data type. For example, there are more patients with RNA microarray data available in the database than with RNA sequencing data. Second is the inclusion criteria of the study. Some studies may specify additional requirements for inclusion of patients, such as age, molecular subtype, and receiving treatment. Third is the analysis type. Since not all clinical variables are available in all patients, if a study performed multivariable analysis with a molecular marker, age, sex, extent of resection and chemotherapy, there would be missing data leading to a smaller number of patients available. Therefore, studies that used the same genomic resource without additional selection criteria performing univariable analysis should have the same number of patients in this analysis. We have clarified this in the methods section (Methods section under “Summary statistics”). It now reads: “*To assess the reproducibility of cohort selection from TCGA, we summarised the number of patients in studies published between 2017-2020 using TCGA RNA microarray. These studies should have the same number of patients because they all used the same RNA microarray dataset from TCGA when there was no further accrual of patients.*”.

The authors should demonstrate the concept of consistency between studies in terms of some biomarkers using examples and quantifiable/qualifiable metrics.

**Response**: The concept of consistency between studies is important as it maintains the quality and allows for reproducibility of studies. The full description and discussion of consistency is beyond the scope of this review, but we now have expanded on our discussion and added relevant references to guide readers on this topic (<https://doi.org/10.1097/ACM.0000000000002351>, <https://doi.org/10.1038/d41586-021-02486-7>) (Discussion section under “Reproducibility”). This review did not set out to present quantifiable metrics of consistency – we stated our *a priori* plan to have no meta-analysis of results extracted from the included studies. Consistency can be described using measures of heterogeneity in a meta-analysis. But for this to be appropriate, studies must be comparable in design. Unfortunately, we were unable to do this because the studies were not comparable. We have now modified our discussion to highlight these aspects (Discussion section under “strengths and limitations”).

Also, what is the impact of these inconsistencies? Is there a way to judge which study to trust in case of such inconsistency?

**Response**: The impact of the inconsistencies described in our manuscript is out of the scope of our review because it requires longitudinal follow up of how the results from these studies were used to develop new therapies for glioblastoma. However, in the context that >70% of researchers were unsuccessful in reproducing another scientist’s experiments (<https://www.nature.com/articles/533452a>), there is a concern with scientific rigour and integrity. Unfortunately, we are unable to recommend a strategy for identifying the preferred study in case of inconsistency. This is because the decision should be based on design of preclinical experiments (including validity of preclinical models, internal validity, mitigation of bias), reporting of preclinical experiments detailing methods to allow for reproducibility, transparency of clinical validation method, method of survival analysis, and reporting of clinical validation. Our review addresses the latter two aspects of this decision-making process. This review encourages researchers to optimise research conduct and reproducibility so there is sufficient transparency for readers to decide what is trustworthy.
